# Acceptability, perceived reliability and challenges associated with distributing HIV self‐test kits to young MSM in Uganda: a qualitative study

**DOI:** 10.1002/jia2.25269

**Published:** 2019-04-01

**Authors:** Stephen Okoboi, Adelline Twimukye, Oucul Lazarus, Barbara Castelnuovo, Collins Agaba, Muloni Immaculate, Mastula Nanfuka, Andrew Kambugu, Rachel King

**Affiliations:** ^1^ Infectious Diseases institute College of Health Sciences School of Medicine Makerere University Kampala Uganda; ^2^ Clarke International University Kampala Uganda; ^3^ The AIDS Support Organization (TASO) Kampala Uganda; ^4^ University of California, Global Health Sciences San Francisco CA

**Keywords:** HIV self‐testing, MSM, peer leaders, HIV testing, TASO, perception and feasibility, Uganda, Africa

## Abstract

**Introduction:**

HIV self‐testing is a flexible, accessible and acceptable emerging technology with a particular potential to identify people living with HIV who are reluctant to interact with conventional HIV testing approaches. We assessed the acceptability, perceived reliability and challenges associated with distributing HIV self‐test (HIVST) to young men who have sex with men (MSM) in Uganda.

**Methods:**

Between February and May, 2018, we enrolled 74 MSM aged ≥18 years purposively sampled and verbally consented to participate in six focus group discussions (FGDs) in The AIDS Support Organization (TASO Masaka and Entebbe). We also conducted two FGDs of 18 health workers. MSM FGD groups included individuals who had; (1) tested greater than one year previously; (2) tested between six months and one year previously; (3) tested three to six months previously; (4) never tested. FGDs examined: (i) the acceptability of HIVST distribution; (iii) preferences for various HIVST distribution channels; (iv) perceptions about the accuracy of HIVST; (v) challenges associated with HIVST distribution. We identified major themes, developed and refined a codebook. We used Nvivo version 11 for data management.

**Results:**

MSM participants age ranged between 19 and 30 years. Participants described HIVST as a mechanism that would facilitate HIV testing uptake in a rapid, efficient, confidential, non‐painful; and non‐stigmatizing manner. Overall, MSM preferred HIVST to the conventional HIV testing approaches. Health workers were in support of distributing HIVST kits through MSM peers. MSM participants were willing to distribute the kits and recommended HIVST to their peers and sexual partners. They suggested HIVST kit distribution model work similarly to the current condom and lubricant peer model being implemented by TASO. Preferred channels were peers, hot spots, drop‐in centres, private pharmacies and MSM friendly health facilities. Key concerns regarding use of HIVST were; unreliable HIVST results, social harm due to a positive result, need for a confirmatory test and linking both HIV positive and negative participants for additional HIV services.

**Conclusions:**

Distribution of HIVST kits by MSM peers is an acceptable strategy that can promote access to testing. HIVST was perceived by participants as beneficial because it would address many barriers that affect their acceptance of testing. However, a combined approach that includes follow‐up, linkage to HIV care and prevention services are needed for effective results.

## 1 Introduction

In 2017, 21 million people were estimated to be on ART, of which 7.6 million (36.2%) reside in Sub‐Saharan Africa 1. Despite this progress in the uptake of ART and HIV testing services 1, 2, 3, 4, 5, UNAIDS estimated that 35% of all PLWH globally do not know their HIV status in 2017 1, 2.

New HIV infections in Uganda are now concentrated among key populations particularly sex workers and men who have sex with men (MSM), accounting for more than a third of the new HIV infections 6, 7, 8. An effort to strengthen prevention and increase availability of HIV testing in Uganda has been scaled up to key populations; however, testing rates remain low in MSM 9 who also have limited access to facility‐based HIV testing due to gay‐related stigma, discrimination and legislation 10, 11. Currently, in Uganda, HIV testing for key populations like MSM who have limited access to HIV prevention services is being scaled up through hotspots, mobile outreaches and moonlight testing approaches. A “moonlight testing approach” is HIV testing during evening hours in high‐risk settings such as MSM hotspots 9. Linkage to care for those who test HIV positive and initiation onto pre‐exposure prophylaxis for MSM at high risk is a strategy being strengthened using peer facilitators 9.

HIV self‐testing (HIVST) is a flexible, accessible and acceptable emerging technology that could increase access to HIV testing through social and sexual networks and empower partners of unknown HIV status to know their HIV status 12. In 2018, an oral fluid‐based rapid HIV‐1/2 antibody test (Ora‐Quick) was recommended by the Uganda Ministry of Health for use in public and private facilities 9. HIVST use has been found acceptable for MSM in South Africa, USA, China and Australia and can increase testing uptake and frequency, potentially improving early detection of HIV among MSM and their networks. The reported high acceptability has been associated with confidentiality, ease of access and privacy, with concerns relating to capability in performing the HIVST, and the need for HIV counselling support, linkage to care and follow‐up [13, 14, 15, 16, 17, 18, 19, 20, 21].

We hypothesized that using peer‐to‐peer links may be particularly suitable for a new device such as HIVST for Ugandan MSM in a highly stigmatized environment 10, 11. Peers mirror the targeted individuals in terms of their experience, status or social role, and other aspects that define “association,” that are thought to provide support and encouragement for behaviour change in ways that non‐peers cannot provide. Peer‐to‐peer links have potential for decreasing stigma; increasing social support, empowerment; access to health information, STI and HIV services hence potentially higher numbers of MSM tested and linked to HIV prevention and care services 10.

We conducted a qualitative formative study to assess the acceptability of HIVST kit distribution, perceived reliability and challenges associated with using MSM peers to distribute HIVST kits to their sexual networks in order to scale‐up HIV testing to MSM in a semi urban Ugandan setting and to identify optimal distribution channels for future HIVST scale‐up 12, 13, 22, 23, 24, 25, 26, 27, 28, 29, 30. This approach is embedded within implementation science research seeking to translate and implement research evidence into practice.

## 2 Methods

### 2.1 Study design

This was a cross‐sectional study that employed qualitative data collection methods among MSM and health workers from March to May, 2018 at The AIDS Support Organisation (TASO) in Masaka and Entebbe, Uganda. We enrolled MSM aged ≥18 years in the community who were invited by MSM peers linked to care and healthcare workers experienced in working with MSM. Participants verbally consented to participate in the eight FGDs (six MSM and two health workers groups).

### 2.2 Study participants and selection

A purposive sample of 74 adult MSM and 18 healthcare workers from Entebbe and Masaka were selected from a list of eligible participants. Criteria for selecting study participants included male MSM who: (1) had been identified by peers; (2) were aged 18 years and older; (3) who had anal sex with men in the past year; (4) four different HIV testing history categories as described below. For healthcare providers, criteria were selecting different cadres who were previously trained as dedicated liaison healthcare providers for MSM clients at TASO centres. Peers identified and invited the MSM in the community for study interviews at TASO centres or neutral venues agreed upon by participants. FGDs were scheduled at a time convenient to the participants. The MSM FGD participants were from four HIV testing categories of the population of interest including: (1) HIV tested >1 year previously; (2) tested between six months and a year before enrolment; (3) tested, three to six months previously; (4) never tested. TASO Health workers categories included; pharmacy technicians, laboratory technologists, counsellors, client's relations officers, linkage officers and nurses.

### 2.3 Data collection

We conducted eight FGDs (six with selected MSM and two with health workers at TASO‐ Masaka and Entebbe (eight to twelve participants per group). Our FGDs sought to elicit information on; acceptability, perceived reliability and challenges associated with using and distributing HIV self‐test kits and on how best to identify peers, how to share information among peers, how to distribute HIVST kits, how to link tested MSM to a health facility for confirmation of an HIV test result. Ora‐Quick oral fluid‐based second generation HIVST kits (OraQuick^®^ Rapid HIV‐1/2 Antibody Test from Orasure Technologies, Inc. Bethlehem, PA, USA) were demonstrated for participants who were then asked to comment on the design of the kit and the testing procedures. The sensitivity and specificity of the HIVST kit were explained by the facilitator using a lay language, if the participant asked. We used an FGD guide for both MSM and healthcare workers with semi‐structured questions based on the current literature on acceptability, barriers to and facilitators of using peer navigators, to scale‐up HIVST. We assessed perceptions and acceptance on: (i) the use of existing HIV testing services; (ii) the acceptability of HIVST; (iii) preferences for various HIVST distribution channels (e.g. facilities, vendors, peers); (iv) perceptions about the accuracy of HIVST; (v) linkage to care issues; and (vi) barriers to HIVST that may be group specific. Each FGD lasted approximately 60 to 90 minutes. We developed vignettes to serve as triggers for discussions.

FGDs were conducted in English for health workers and Luganda (the local language spoken in the region) for MSM. All interviews were audio recorded. Study tools and demonstration instructions were translated into Luganda, the main language spoken in the catchment area of the study participants. Luganda audio recordings were translated and transcribed into English. All transcripts were typed in a word document. Data were collected by three experienced social scientists trained in FGD moderation and note taking. The staff received Good Clinical Practice training as well as specific training on conducting FGDs, the study protocol and the FGD guide.

### 2.4 Data analysis

Data analysis focused on acceptability, perceived test reliability and challenges associated with HIVST kits distribution using selected MSM peers to distribute HIVST testing kits to fellow MSM which was the major domain of interest. We identified major themes and developed a codebook to describe each category. We used Nvivo version 11 for data management as well as coding according to domains of interest and our theoretical foundation.

AT (Social scientist), SO (Principal investigator) and RK (Supervisor) were involved in data analysis. These research team members read all the transcripts and identified general themes and categories. We used an inductive approach to the data by analysing it through coding and then identifying themes. We discussed the themes in a group and then developed explicit summaries describing each category and themes. The framework for analysis was developed based on preliminary analysis of transcripts and informed by the topic guides. In the next step, we sorted quotes from the transcripts based on their thematic similarities using Nvivo. Quotations and key phrases are highlighted in the findings.

### 2.5 Ethical approval

This study was approved by Infectious Diseases Scientific Review committee, TASO Research Ethics Committee, and the senior management of TASO, The University of California, San Francisco, the National Council for Science and Technology. The study purpose and procedures were explained to all participants. Those who agreed to take part in the study provided verbal consent, consistent with the guidelines from the regulatory bodies concerned about the criminalization of MSM in Uganda 31.

## 3 Results

### 3.1 Socio‐demographic characteristics

MSM participants were aged between 19 and 30 years while health workers were between 25 and 46 years.

### 3.2 HIVST perceived as beneficial

Participants perceived HIVST as beneficial because it would reduce most of the current perceived and experienced barriers to HIV testing such as distance to HIV testing facilities, long waiting times at facilities, lack of MSM friendly testing services, stigma and discrimination that affects MSM's access to and acceptance of HIV testing.

Some like me ‐ I might not test even when I want to test because I fear doctors to know I am MSM, but I can test if am alone, I can have that courage to test. (MSM, tested >1year, TASO‐ Masaka)

When you look at the MSM, they are not patient at the service centres. They don't want to go to a service centre, and wait for the process to be conducted as they wait for their results. But if an opportunity comes and they get access to the testing kits, it will be an opportunity for them to test themselves from home without going to health facilities to wait. (Health worker, TASO‐ Masaka)

Most interviewees perceived HIVST distribution through peer networks as efficient and accessible. What they requested was the availability of the HIVST kits through MSM peers so that they could easily access the kits to conduct self‐testing in private houses and locations.

Currently, there are no specialized HIV testing services for MSM in most health facilities and our participants did not feel comfortable in general clinics.

It is personal. I don't have to go and line up in a hospital, I am alone, I wait for my results, it is easy for me… even the fact that we are very secretive, it Is all about secrecy, so it is easy for me to use it in my private room, no one needs to know. (MSM, never tested Masaka)

In addition, participants in all groups noted that HIVST would reduce costs and time. The majority expected to receive free self‐test kits, however some suggested setting affordable prices for self‐test kits that would be distributed in private pharmacies or clinics.

I would actually use HIVST kit, because it saves me the time where I find a long line, I can try it out in my house, I can go to confirm the results having tested from home. It is efficient and it's close to you. (MSM, mixed group, TASO‐ Entebbe)

Participants described HIVST as a mechanism that would increase HIV testing in a rapid, efficient, confidential and non‐painful and non‐stigmatizing manner.

It is very convenient for them because it caters for privacy and confidentiality and time saving and I look at it to increase the uptake of HIV among the MSM. So, they will welcome it. (FGD, health worker, TASO‐ Entebbe)

Participants reported that HIVST enhances couple HIV testing. MSM couples who fear going to the hospital can receive HIV results and know together through HIVST distribution strategy to sexual networks. This promotes mutual disclosure and enhances risk reduction.

Like the other one said, when his sexual partner doesn't want to go for testing, it can help them to test themselves so that they don't go to the hospital for testing. And then they will get to know each other's results. (FGD, MSM, tested >1 year, TASO‐ Masaka)

### 3.3 Reliability of self‐testing results is a key issue among MSM

Among MSM and some health workers, we observed mixed reactions about the reliability of HIVST results. Some participants believed HIVST will give reliable results however, they were quick to say that this is dependent on following the correct testing procedures and instructions.

There is nothing which is not possible unless you don't use it as instructed. Me, I believe it can give me true results if I follow instructions. (MSM, tested >1 year TASO‐ Masaka)

Inaccuracy of results according to the MSM participants would be a result of lack of information by the user of the self‐test kit. The issue of translating the HIVST instruction manual to appropriate languages was considered important for information sharing.

The accuracy will depend on the information/awareness of the person who is going to carry out the HIV self‐testing. But if the information is wrongly given then accuracy may not be achieved. (MSM, never tested group TASO‐Masaka; and Health worker, TASO‐Entebbe)

One participant noted that previously in school he was taught there was very little HIV found in saliva, so that an HIV test based on saliva may not give accurate results.

What I know since I studied science from primary to high school and we were taught that to get HIV in saliva you have to get 20 litres to detect HIV in it. So, I am seeing it as a myth and something that is not real and fake. (MSM, mixed group‐TASO‐Entebbe)

### 3.4 Peer HIVST kits distribution strategy is highly acceptable among MSM population

**Figure 1:**
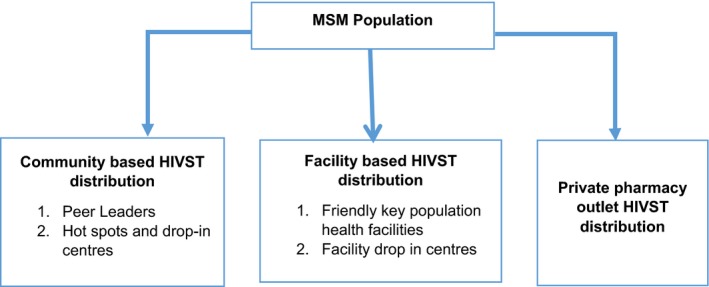
Preferred HIV self‐test distribution channels among men who have sex with men

The HIVST preferred distribution channels were through MSM peers, MSM hot spots, LGBTQ communities, friendly MSM HIV facilities and private pharmacies. The majority of participants suggested that peer leaders distribute self‐testing kits to fellow MSM because they easily link with the providers and are trusted by peers. Participants reported that they felt that the peer‐to‐peer approach to distribute the self‐test kits will enhance access to HIV testing since MSM face increasing levels of stigma and discrimination even within health facilities. Some participants emphasized that peers have to be in good terms with fellow MSM and have the ability to communicate about the benefits of self‐testing as well as the ability to clearly explain self‐testing procedures and instructions (Figure 1).

I would recommend these HIV self‐testing kits to be given to peers because with peers they can be accessible or reached at any time of the day 24/7 we can get in contact with them and get the services. Peers would be the number one priority, grassroots organization the second, health facility the third and hot spot centres the last. (MSM, mixed group, TASO‐ Entebbe)

To me, a peer always has people that trust him, so I think if he is a peer distributing the test kits, it can be good for me then he will be the one to refer me to hospital (MSM, tested 3‐6 months, TASO‐ Entebbe)

### 3.5 Use of health workers to distribute HIVST Kits

Some participants suggested distribution of self‐test kits through health facilities that offer HIV services. This is on condition that they do not have bias towards the gay community, and they offer friendly services to MSM. This was because they trusted health workers to issue reliable kits, and they would easily follow them up in case test results turned out HIV positive (Figure 1).

The easiest place would be friendly health facilities but sometimes these places aren't open all the time. You might come and there are no people and the place is locked, for example during weekends. (MSM, tested 3‐6 Months, TASO‐ Masaka)

### 3.6 Use of private pharmacies to distribute HIVST Kits

There was a consideration of private pharmacies as an essential place to distribute self‐test kits. This was mentioned particularly for MSM who wanted extra privacy. In addition, the general fear of stigma associated with HIV testing centres was noted. MSM presumed that no one would point a finger at them if they approached any big pharmacy for a self‐test kit. In addition, the pharmacies would be open longer hours than health facilities. (Figure 1).

I am saying some people fear coming to TASO because people might say that they are HIV positive. There are such people. Now, it would be good if it (HIVST) is put in big pharmacies as well. (MSM, tested >1 year TASO‐ Masaka)

### 3.7 Social harm; a key issue in acceptability of HIVST

The MSM and health workers had similar views about challenges of HIVST such as: social harm to individuals due to lack of counselling after receiving positive results, and what mechanism would work best to link those testing HIV positive to care. Participants feared that people may resort to suicidal tendencies after testing HIV positive. Others could develop negative reactions such as anger and bitterness (Table 1).

People have been emphasizing HIV counselling and testing, and pre‐test counselling is really key; I don't know really whether now HIV is becoming so normal that someone has the courage actually to test positive and they don't, resort to committing suicide? Because I am actually worried on that side, breaking the news to myself in my room. Am seeing that as a really a big issue to think about. (Health worker, TASO‐ Masaka)

Lack of psychosocial support was mentioned as a major risk of HIVST. Some MSM may fail to accept and cope with positive results since there is no professional counsellor to offer post – test counselling.

Among those who test HIV positive, how will they cope with the test result when there is no counselling; we used to test with a counsellor, at least if am found reactive there is a way how he or she is going to handle me because he/she is experienced. (MSM, tested 3‐6 months, TASO‐ Masaka)

**Table 1:** Emerging themes specific to HIVST uptake among MSM

Characteristic of the subject	Emerging issue	Results	Strategies proposed to ease scale‐up
Peer level dynamics	Peer influence and MSM involvement in HIVST	Barriers MSM suffer stigma and discrimination Facilitators HIVST acceptable by MSM Will increase numbers of MSM tested for HIV hence identification of MSM who are HIV positive HIVST distribution can be integrated with condoms and lubricant distribution	Integrate HIVST within key populations programmes Train peer leaders in HIVST and basic counselling
Socio‐economic status of MSM	Social space for MSM in hot spots and drop in centres	Barriers Lack of time to seek facility‐based testing Long distance to the hot spots Lack of enough HIVST, mostly used by demonstration projects only Facilitators Free association with peers at hots spots and drop in centres Good communication skills increases HIVST acceptance and linkage to care	Increase community empowerment to avoid fear of health facilities by the MSM Increase access to healthcare information and HIV services to the MSM Provide less stigmazing and non‐discriminating care to MSM
HIV related Stigma	Stigma hinders access to HIV testing among MSM	Barriers MSM seen as minority and not cultural accepted Facilitators Peer network among MSM seen as a mechanism to distribute HIVST Many preferred HIVST distribution channels	Train more peer leaders to strengthen role of peer approach in HIVST kits distribution Avail incentives (transport, airtime) to peers to distribute HIVST Ensure availability of HIVST
MSM knowledge about HIVST	Social influence social identity, likes and dislikes of HIVST, knowledge about other HIV preventive measures and access to treatment for those who test HIV positive	Barriers MSM negative attitude to health facility Fear of social harm due to HIV positive result MSM perception that they cannot be infected with HIV Facilitators Peer support mechanism already available Willingness of the peers to be trained	Increase HIVST awareness Train peer leaders in basic HIV counselling and linkage skills to support follow‐up Train community leaders about HIVST
MSM Knowledge about HIVST testing procedures	Demonstrability	Barriers Fear of the HIVST not a confirmatory test Accurate interpretation of testing results Facilitators Willingness to attend kits demonstration by the trainers Availability of a short HIVST demonstration video that has been developed.	Develop and provide HIVST information brochures in locally appropriate languages
Accessible distribution points and approaches	Observability Access to hot spots for HIVST, Availability of peers to scale‐up HIVST MSM friendly clinics Provide incentives to motivate peers	Barriers HIVST is relatively new technology and concept Peers not trained in HIVST procedures and counselling package Facilitators Availability of MSM peers who have influence to fellow MSM Peers willing to be trained and distribute the kits	Train peers and sensitize the populace about HIVST technology Train health care providers in HIVST Avail incentives to peers and ensure availability to HIVST kits
Legal framework and policies on HIVST in Uganda	Ensure accessibility of HIV testing services to key and priority populations	Barriers Unfavourable legal framework for MSM Limited training on HIVST testing guidelines Facilitators HIVST testing kits to be freely distributed to all	Scale‐up HIVST training to health workers and peers Strengthen legal framework at local/national level regarding HIVST uptake and clearly define the target group

MSM, men who have sex with men; HIVST, HIV self‐test.

## 4 Discussion

We found positive, negative and conflicting perceptions and attitudes towards the acceptability of HIVST kits and distribution strategies. Distribution by MSM peers was highly acceptable to both MSM and healthcare workers. MSM cited access, cost, convenience and confidentiality as key benefits of distribution through peer networks. Concerns about legislation against MSM, stigma and about the potential for a positive diagnosis were commonly cited drawbacks (Table 1). These concerns are potentially generalizable to many African countries where being gay is illegal. Our findings agree with previous studies in UK, USA, China, South Africa and Australia indicating that peer distribution of HIVST kits is acceptable among MSM because of its associated confidentiality, ease of access and privacy, with concerns relating to capability in performing the HIVST, and the need for HIV counselling support and follow‐up 13, 14, 15, 16, 17, 18, 19, 20, 21. We also found that for the peer distribution strategy to remain highly acceptable, programmes should develop intervention strategies such as education and sensitization of MSM about HIVST that address the fears around accuracy and social harm. In addition, having a robust peer training and follow‐up strategy while re‐emphasizing importance of confirmatory testing for those who test positive and access to treatment including HIV prevention measures like condom use and PrEP are crucial.

MSM reported that HIVST distribution through peer networks will motivate them for more frequent HIV testing because it is convenient, with easy access and ensures confidentiality. This finding could promote the achievement of the UNAIDS first 90 target, and encourages policy developers, advocates, HIV programme managers and healthcare workers who have been trying to ensure that populations at greater risk of HIV infection test more frequently 16. The requirement to test within the healthcare setting in Uganda including non‐governmental organizations providing care for people living with HIV is problematic for many key populations including the MSM who fear the punitive laws in the country. Our results highlight that if the concerns of the MSM community are considered and solutions found, peer‐distribution of self‐testing kits in a high prevalence country like Uganda could be one effective option to increase access to HIV testing among vulnerable and stigmatized groups like MSM.

We also observed that HIVST might increase self‐care since the HIV testing can be done remotely or even integrated within other MSM specific care programmes. The integration of HIVST within HIV care programmes will be driven by public health policies that aim to increase the variety of HIV testing options 17, 18. Consistent with the literature 19, 20, 21 we observed that MSM reported that they would be more likely to use HIV‐self testing kits as a key primary HIV testing method. This is mainly because of greater confidentiality, increased cost‐effectiveness, less invasive and the increased preference for HIVST. It is paramount that programmes design HIVST interventions as an option which increases access for MSM to test more frequently. We should also ensure development and testing of innovative strategies to integrate HIVST within the existing HIV testing pathways. In particular, it may be highly acceptable if HIVST is integrated with the MSM HIV care programmes including the use of peers.

## 5 Strengths and limitations

This is the first report on formative qualitative results studying young MSM HIVST distribution strategies, perceived HIV test results reliability and perceptions of HIVST in a Uganda MSM population. Our data will be informative when considered alongside other published literature that reports HIVST kits distribution as acceptable among MSM and healthcare workers 15. Furthermore, as a study which is qualitative in nature, these findings should be understood as indicative of the diversity of values and preferences and their meanings among MSM in Uganda. However, this data may not be representative of all the MSM and those who are not MSM. This is because we purposively selected MSM participants through their peer leaders and healthcare workers who were dedicated MSM care providers linked to a non‐government organization and not a public healthcare system. We describe how the context of implementation could shape the design and delivery of future HIVST kits services to the hard to reach key populations.

## 6 Conclusions

Distribution of HIVST kits by MSM peers is an acceptable strategy that can promote access to HIV testing. HIVST was perceived by MSM and health workers as beneficial because it would address many barriers that affect their acceptance of testing. However, a combined approach that includes follow‐up, linkage to care and prevention services is needed for effective results.

## Competing interests

The authors declare that they have no competing interests.

## Authors’ contributions

SO, BC and RK designed the study. SO, CA, NM, IM and LO supervised and collected the study data. AT, SO and RK conducted or contributed to data analysis. AK, SO, RK, BC, CA and LO interpreted the data. SO prepared the original manuscript. RK, AT, NM, AK, and BC contributed to subsequent revisions. All authors read and approved the final manuscript.
